# Constant up-regulation of BiP/GRP78 expression prevents virus-induced apoptosis in BHK-21 cells with Japanese encephalitis virus persistent infection

**DOI:** 10.1186/s12985-015-0269-5

**Published:** 2015-02-26

**Authors:** Hey Rhyoung Lyoo, Soo Young Park, Ji Young Kim, Yong Seok Jeong

**Affiliations:** Department of Biology, College of Sciences, Kyung Hee University, Seoul, 130-701 Republic of Korea

## Abstract

**Background:**

Persistent infection of the Japanese Encephalitis Virus (JEV) has been reported in clinical cases, experimental animals, and various cell culture systems. We previously reported the establishment of spontaneous JEV persistent infection, assisted by defective interfering particle accumulation and/or attenuated helper viruses, in BHK-21 cells devoid of virus-induced apoptosis, cBS6-2 and cBS6-3. However, cell-specific factors may play important roles in controlling JEV replication and have never been assessed for this specific phenomenon. Recent evidence suggests that viruses have evolved various mechanisms to cope with endoplasmic reticulum stress signaling pathways for their efficient amplification and transmission, including the unfolded protein response (UPR).

**Results:**

To identify the host cell factors that affect JEV persistence, we investigated the expression of essential UPR factors in cBS6-2 and cBS6-3 cells. Of the selected UPR factors tested, the most noticeable deviations from those of the normal BHK-21 cells with JEV acute infection were as follows: the suppression of C/EBP homologous binding protein (CHOP) and the constant up-regulation of immunoglobulin binding protein (BiP) expression in cBS6-2 and cBS6-3 cells. In JEV acute infection on normal BHK-21 cells, silencing CHOP expression through specific siRNA blocked cell death almost completely. Meanwhile, depletion of BiP by specific siRNA unlocked CHOP expression in cBS6-2 and cBS6-3 cells, resulting in massive cell death. Fulminant apoptotic cell death for both cell clones on tunicamycin treatment revealed that the JEV persistently infected cells still contained functional arms for cell fate decisions.

**Conclusions:**

BHK-21 cells with JEV persistent infection strive against virus-induced apoptosis through constant up-regulation of BiP expression, resulting in the complete depletion of CHOP even with apparent virus amplification in the cells. Accordingly, the attenuation of virus replication as well as the modifications to cell metabolism could be additional factors contributing to the development of JEV persistent infection in mammalian cells.

**Electronic supplementary material:**

The online version of this article (doi:10.1186/s12985-015-0269-5) contains supplementary material, which is available to authorized users.

## Introduction

Viruses have evolved a wide range of strategies to persist in their hosts. It remains a challenge to understand the mechanisms whereby viral persistence is established and maintained, especially viral persistence within a cell or group of cells. Mechanisms by which RNA virus persistence is initiated and maintained usually involve two virus-specific factors: the generation of defective interfering (DI) particles or temperature-sensitive mutation of wild-type virus [1,2]. Research suggests that host factors involved in the control of persistent infection relate to elements of innate immunity in Morbillivirus [3] and cellular protein synthesis in Reovirus [4].

Protein synthesis and folding occurs in the endoplasmic reticulum (ER). Mammalian cells have evolved many sophisticated signaling pathways to monitor any abnormality, including the accumulation of misfolded proteins; these pathways are known as the unfolded protein response (UPR) [5]. These signaling pathways monitor the ER’s capacity to refold and/or remove abnormally folded proteins and to make cell-fate decisions according to the homeostatic balance [6,7]. In all known animal cells, the following are known to be activated to initiate the UPR: three ER-localized transmembrane UPR transducers, inositol requiring kinase 1 (IRE1), double-stranded RNA-activated protein kinase-like kinase (PERK), and activating transcription factor 6 (ATF6) [8]. Under basal conditions, these three sensors are associated with immunoglobulin binding protein (BiP), also known as GRP78, which is a chaperone of the heat shock protein 70 family. Each branch operates parallel with a particular target downstream and contributes to both cell-protective and cell-death pathways [6,7]. Under severe or chronic ER stress, the UPR switches its mode of action toward apoptosis. C/EBP homologous binding protein (CHOP), also known as growth arrest and DNA damage-inducible protein 153 (GADD153), is the pro-apoptotic transcription factor that plays an important role in regulating cell death after ER stress [9,10]. Several molecular mechanisms of CHOP-induced apoptosis have been cited, such as compromised alteration of Bcl-2 family proteins [11,12].

A variety of viruses induce ER stress and the UPR, having evolved various mechanisms to cope with the UPR [13]. West Nile virus modulates all three arms of the UPR and induces numerous apoptotic responses, including induction of CHOP expression [14]. Modulation of the UPR by the West Nile virus is regulated differentially along with its replication cycle [15]. Similar to other flaviviruses, the dengue virus also induces the three arms of the UPR and CHOP expression. However, activated CHOP does not induce its downstream apoptotic markers, such as suppression of anti-apoptotic protein Bcl-2 and activation of caspase-3 or caspase-9 [16,17]. In addition, studies of the hepatitis C virus have shown that both viral structural (envelope) and non-structural (NS2) proteins can induce ER stress and the UPR activation with up-regulation of BiP and CHOP [18,19].

Japanese encephalitis virus (JEV), a member of the *Flaviviridae*, is the causative agent of encephalitis in humans and can be transmitted by persistently JEV-infected mosquitoes [20]. Viral persistence in the nervous systems of JEV-infected patients has been shown in approximately 5% of JEV cases, suggesting that JEV persistence may contribute to neural sequelae after acute infection [21]. Though JEV is usually cytolytic for susceptible cells, persistent infection of JEV has been established in various cell cultures, including baby hamster kidney (BHK)-21 [22-28] as well as in a mouse model [29]. The underlying mechanisms for JEV persistence in cultured cells are not clearly described. We have previously demonstrated spontaneous establishment of persistent JEV infection in BHK-21 cells via serial undiluted passages without any supplemental treatment [27]. Examples of supplemental treatment include Bcl-2 overexpression [26] or indirect infection with supernatants from persistently JEV-infected mosquito cells [30]. Our previous study suggested that DI particle accumulation and helper virus attenuation are possible mechanisms for the development and maintenance of JEV persistence in BHK-21 cells. Nonetheless, labile cellular factors that may play a role in JEV persistence have never been identified in this system. JEV also induces ER stress and the UPR, and studies suggest that the activation of the UPR is a major cause of JEV-induced apoptosis [31]. The UPR has never been assessed in JEV persistent infection, however.

In this study, we utilized two persistently JEV-infected BHK-21 cell clones (previously reported in [27]) to identify the cell-specific factors of the UPR involved in JEV persistence in mammalian cells. These cell clones seldom, if ever, undergo apoptosis, while JEV replicates actively within. We observed that there was no CHOP expression at any time, but a significant amount of BiP expression was constant in these cells. Knockdown of BiP expression resulted in CHOP induction and subsequent cell death. Because the level of JEV amplification in these cells was not low enough to hold an apoptotic process, we suggest that the readjustment of BiP expression in host cells could be one of key factors involved in cell fate decision under viral persistence.

## Results and discussion

### BHK-21 cells with JEV persistent infection avoided virus-induced apoptosis even with active virus replication

**Figure 1:**
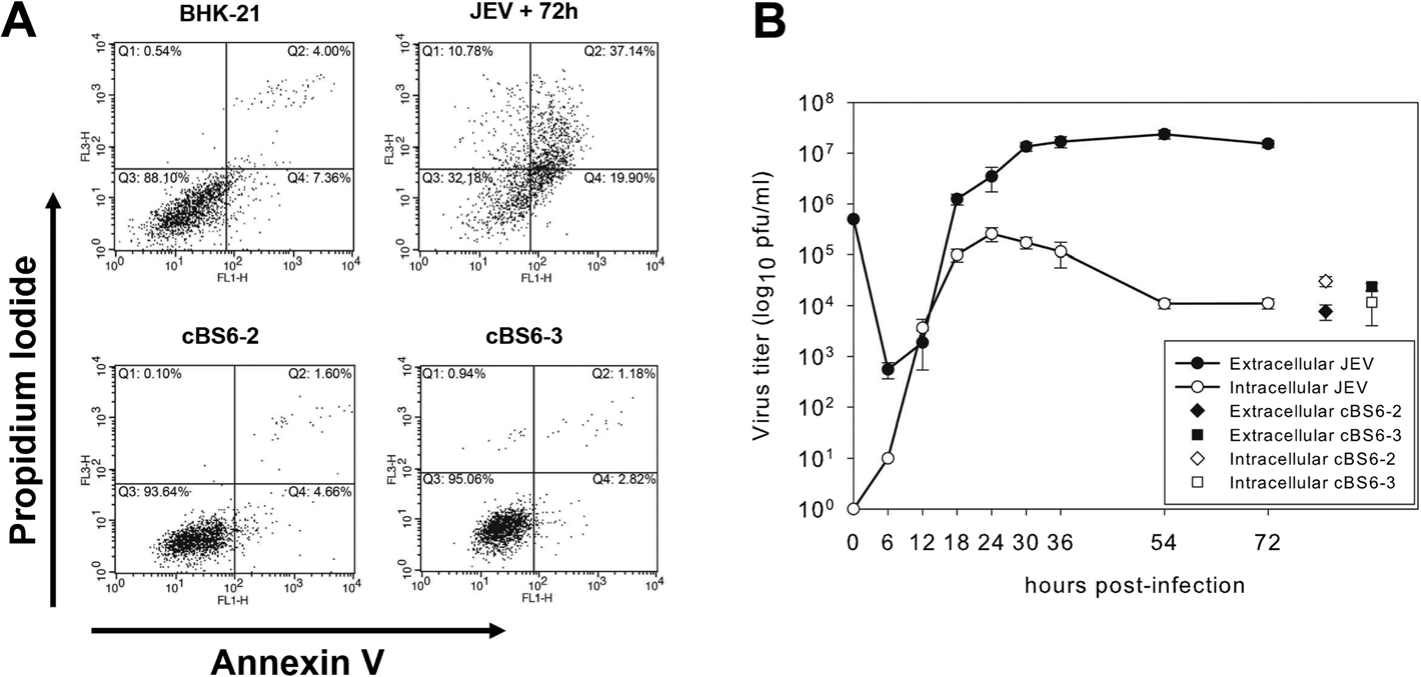
**Persistently JEV-infected cell clones show apoptosis resistance even with active virus replication. (A)** BHK-21 cells either mock-infected or infected with JEV at an MOI of 1 and harvested 72 hr later. PI cell clones were harvested at 72 hr after freshly seeding. Prepared cells were stained with annexin V/propidium iodide, and individual flow cytometric dot plots were displayed. **(B)** The extra- and intracellular virus samples were collected at the indicated p.i. time and counted by plaque-forming assay.

Many viruses that are originally cytopathic have been found to lose their cytopathicity when the persistent infection is established [2]. JEV infection induces severe cytopathic effects in various cell culture systems, including BHK-21 cells, and researchers have documented the ER stress response and subsequent apoptosis in the JEV-infected cells [31,32]. In order to assess how much of the JEV persistently infected BHK-21 cell population is destined to apoptosis while continuously producing infectious virus particles, the two BHK-21 cell clones with JEV persistent infection—cBS6-2 and cBS6-3—were subjected to flow cytometry analysis after annexin V/propidium iodide staining. The number of apoptotic cells and the late apoptotic or necrotic cells increased significantly upon wild-type JEV infection in normal BHK-21 cells (Figure 1A). On the contrary, the number of cBS6-2 or cBS6-3 cells with JEV persistent infection undergoing apoptosis seemed to remain below the basal level shown in the naïve BHK-21 cells. The amount of intracellular infectious JEV particles produced in the persistently infected cells was 5- to 7-fold less than that of an acute infection (Figure 1B). All cells subjected to the flow cytometry were plated and analyzed at the same time points. The cells were grown under the same culture conditions, and no notable difference in the cell numbers of each population was observed.

### Constant expression of elevated amounts of BiP and complete depletion of CHOP were associated with the survival of the persistently infected BHK-21 cells

**Figure 2:**
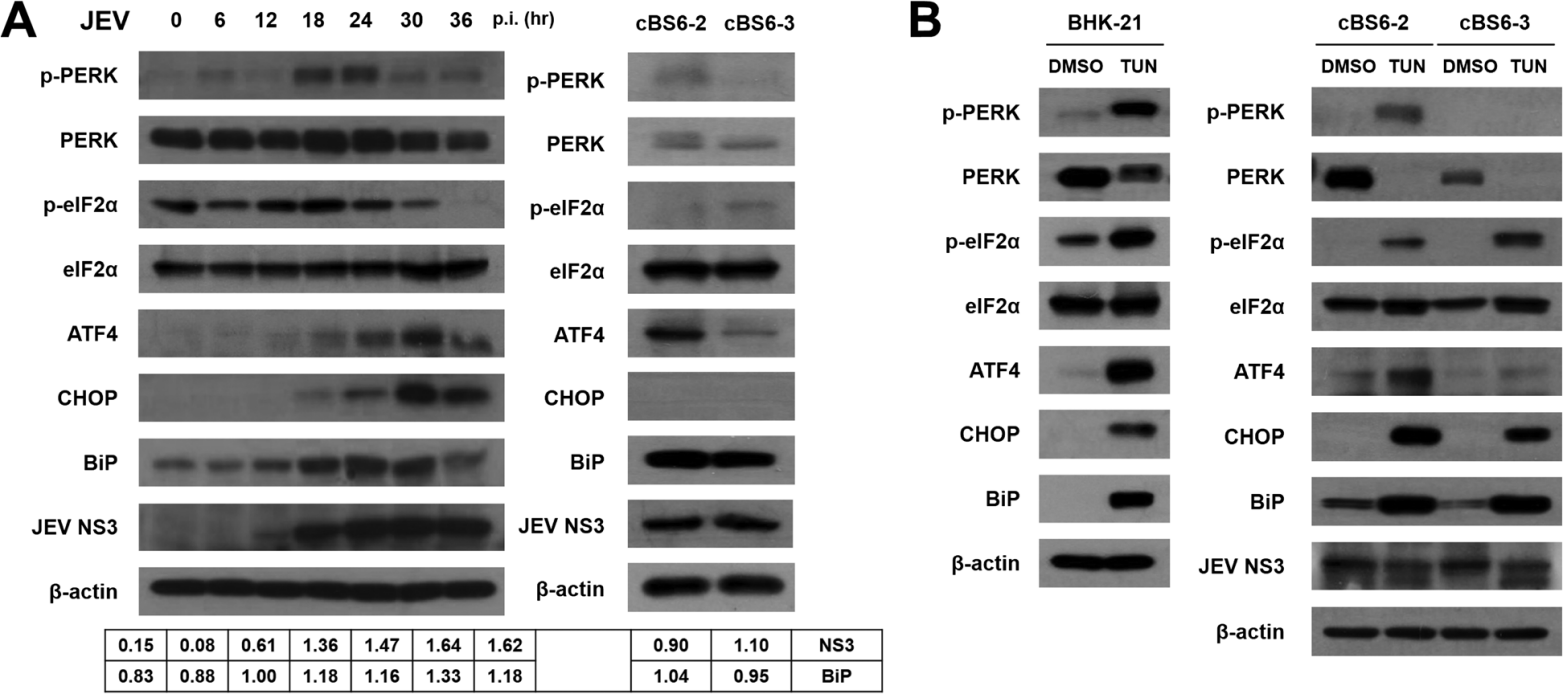
**Modulation of the unfolded protein response in persistently JEV-infected cell clones. (A)** Protein samples were collected from BHK-21 cells infected with JEV at an MOI of 1 at 0 to 36 hr p.i., and from persistently JEV-infected (PI) cell clones cBS6-2 and cBS6-3. Cell lysates were analyzed by Western blotting for p-PERK, total PERK, p-eIF2α, total eIF2α, ATF4, CHOP, BiP, JEV NS3, and the internal control β-actin. Band intensities for BiP and NS3 were determined by densitometry and normalized to those for β-actin. **(B)** BHK-21 cells and PI cell clones treated with 0.5 μg tunicamycin mL^−1^ (lanes TUN), or with DMSO, were harvested 24 hr later, and the cell lysates were prepared and determined for protein expression by Western blotting, as described for panel **A**.

JEV infection is known to induce the UPR in BHK-21 cells through BiP-PERK and/or BiP-IRE1 arms, which is followed by CHOP-mediated apoptosis [31,33,34]. Based on these reports, this study primarily assessed the expression profile of the UPR factors in the BiP-PERK arm of the BHK-21 cells with JEV persistent infection. Normal BHK-21 cells were infected with JEV and harvested at 0, 6, 12, 18, 24, 30, and 36 hr post-infection (p.i.) in order to obtain cell lysates. Two JEV persistently infected BHK-21 cell clones, cBS6-2 and cBS6-3, were freshly seeded and harvested at their confluence on the culture flask; their cell lysates were subjected to Western blotting. Phosphorylation of PERK was gradually increased, peaked at 18 hr p.i., and decreased thereafter in acutely JEV-infected cells (Figure 2A). In persistently JEV-infected cells, however, there was only a minute amount of both PERK and p-PERK. Unlike PERK, the expression of eIF2α in the persistently infected cells seemed enhanced, as shown in normal BHK-21 cells with JEV acute infection at 30–36 hr p.i.; however, the amount of p-eIF2α was barely detectable (Figure 2A).

Phosphorylation of eIF2α often leads to inhibition of protein translation in general, but the translation of ATF4 is promoted by p-eIF2α [35]. This phenomenon was reconfirmed in this experiment as the expression of ATF4 increased gradually 6 hr p.i. in JEV acutely infected cells (Figure 2A). CHOP, the key mediator of ER stress-induced apoptosis, was also induced and accumulated gradually along with the ATF4 activation. In contrast, for the JEV persistently infected cell clones, the expression of CHOP was not detected at all, even in the presence of ATF4 for its transcription (Figure 2A). Unlike cBS6-2, impaired expression of ATF4 in cBS6-3 was repeatedly noticed in several independent experiments. It was noteworthy that a much higher level of BiP was maintained in both JEV persistently infected cells throughout the culture period.

To further investigate whether the JEV persistently infected cell clones kept the UPR pathway intact, naïve BHK-21 cells and the two cell clones with JEV persistent infection were treated with 0.5 μg tunicamycin mL^−1^ for 24 hr. Compared to the DMSO-treated control, both tunicamycin-treated normal BHK-21 cells and cBS6-2 cells showed PERK-eIF2α-ATF4 pathway activation followed by CHOP induction (Figure 2B). Most of the cells treated with tunicamycin, including cBS6-2 and cBS6-3 cells, succumbed to apoptotic cell death within 24 hr (data not shown). Although CHOP was also clearly induced, expression of p-PERK or ATF4 in cBS6-3 cells was not comparable to the normal BHK-21 cells or cBS6-2 cells on tunicamycin treatment for unknown reasons. This observation suggests that cBS6-3 cell clones may utilize another pathway to induce CHOP expression, perhaps involving IRE1 activation. These differences in the activation process of the BiP-PERK arm between cBS6-2 and cBS6-3 imply that individual cells comprising a cell batch with JEV persistent infection could have their own unique modification in cellular physiology to avoid the virus-induced apoptosis.

Taken together, these results suggest that the JEV persistently infected cells avoid fulminant apoptosis by maintaining a constant, highly-elevated level of BiP, which results in the complete suppression of CHOP induction.

### Silencing CHOP expression blocked JEV-induced apoptosis without the serious intervention of viral replication

**Figure 3:**
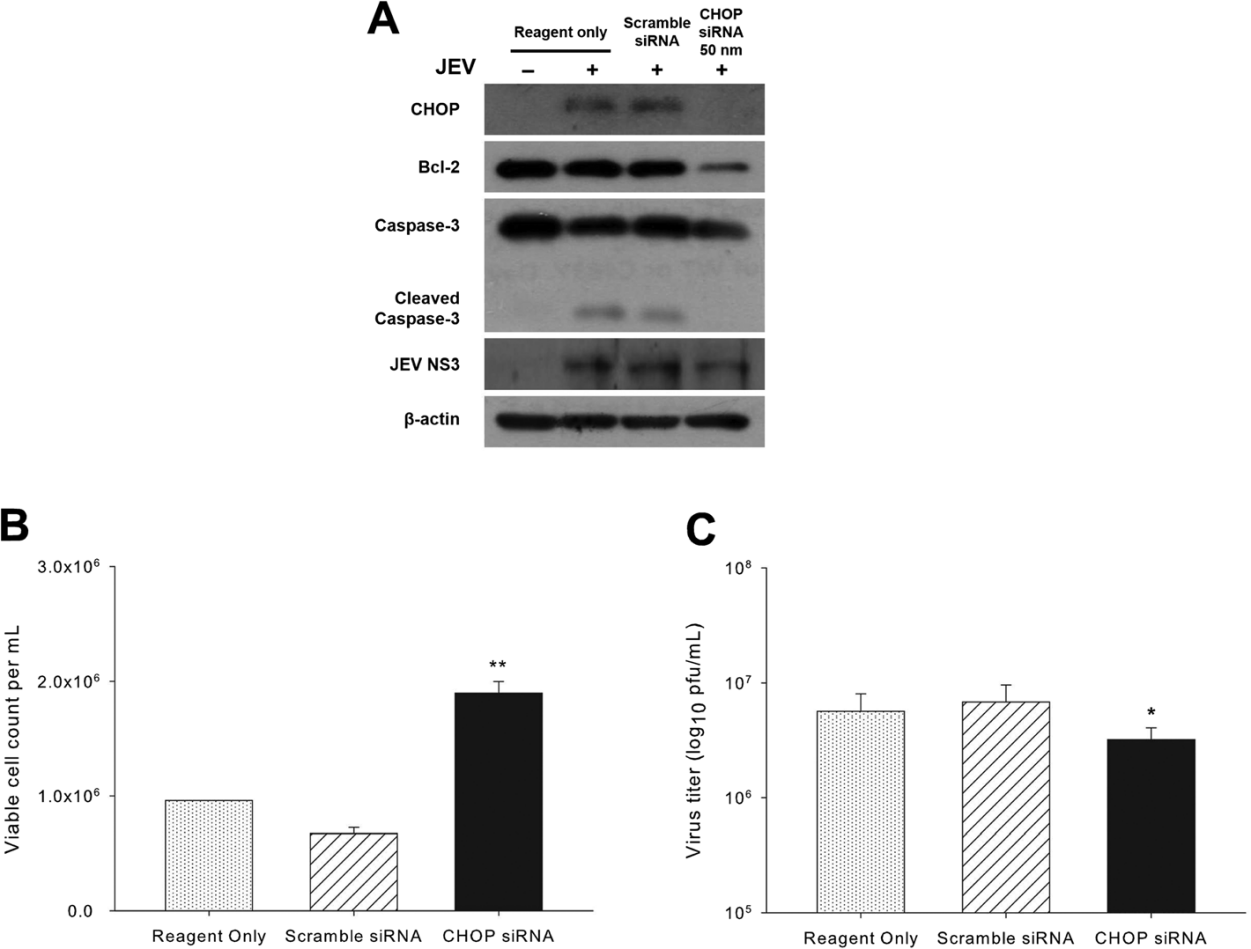
**Effects of CHOP silencing on wild-type JEV infection. (A)** BHK-21 cells were transfected with specific siRNA for CHOP or non-specific scramble siRNA and infected with JEV at an MOI of 1 or mock infected at 24 hr post-transfection. Cells were harvested at 48 hr p.i. The cell lysates were subjected to Western blotting with CHOP, Bcl-2, caspase-3, JEV NS3, and β-actin as an internal control. The number of viable cells **(B)** and infectious virus particles **(C)** from the **(A)** sample were determined by trypan blue exclusion and plaque-forming assay, respectively. *P < 0.05, **P < 0.005.

Based on the observations, there was no detectable CHOP expression during continued virus replication in the JEV persistently infected cells (Figures 1B, 2A, and Additional file 1: Figure S1). Therefore, we attempted to clarify the effects of CHOP on JEV-induced apoptosis and on virus replication efficiency. Naïve BHK-21 cells transfected with specific siRNA for CHOP were infected with JEV at 24 hr post-transfection and harvested at 36 hr p.i. The cell lysates were examined by Western blotting with antibodies against CHOP, Bcl-2, caspase-3, and JEV NS3. The expression of CHOP was efficiently silenced, and cleavage of caspase-3 was not detected in siCHOP-transfected cells (Figure 3A). We also found that, even in the reduced expression of Bcl-2, siCHOP-transfected cells were highly resistant to the JEV-induced cytopathic effect, as measured by trypan blue exclusion (Figure 3A and B).

The level of JEV NS3 protein in the cells transfected with siCHOP and then infected with JEV was slightly lower than that of the other cells transfected with scramble siRNA (Figure 3A). This observation aligns with the result that the virus titer obtained from the CHOP-silenced cells was slightly compromised compared to those from the control cells at 48 hr p.i. (Figure 3C). These results are also consistent with a report noting that up-regulation of CHOP during JEV infection plays a key role in virus-induced apoptosis [31]. As infectious bronchitis virus-induced apoptosis was suppressed and virus replication inhibited in the CHOP-knockdown cells [36], the complete blockage of CHOP expression in cBS6-2 or cBS6-3 cells might intervene in JEV replication and therefore assist in the development of JEV persistent infection. However, a clear explanation for this observation is presently beyond the scope of this study.

### Silencing BiP expression in JEV persistently infected cells led to CHOP induction, followed by severe reduction in cell viability

In this study of JEV persistently infected cells compared to an acute infection in normal BHK-21 cells, the most distinguishable aspects regarding the UPR factors were the complete depletion of CHOP and the constant up-regulation of BiP expression (Figure 2A and Additional file 1: Figure S1). Some previous studies also showed that BiP overexpression attenuates ER stress signaling and is protective against apoptosis [37]. Therefore, we decided to assess the implications of the constant expression of BiP for maintaining cell viability against virus-induced apoptosis in the JEV persistently infected cells.

**Figure 4:**
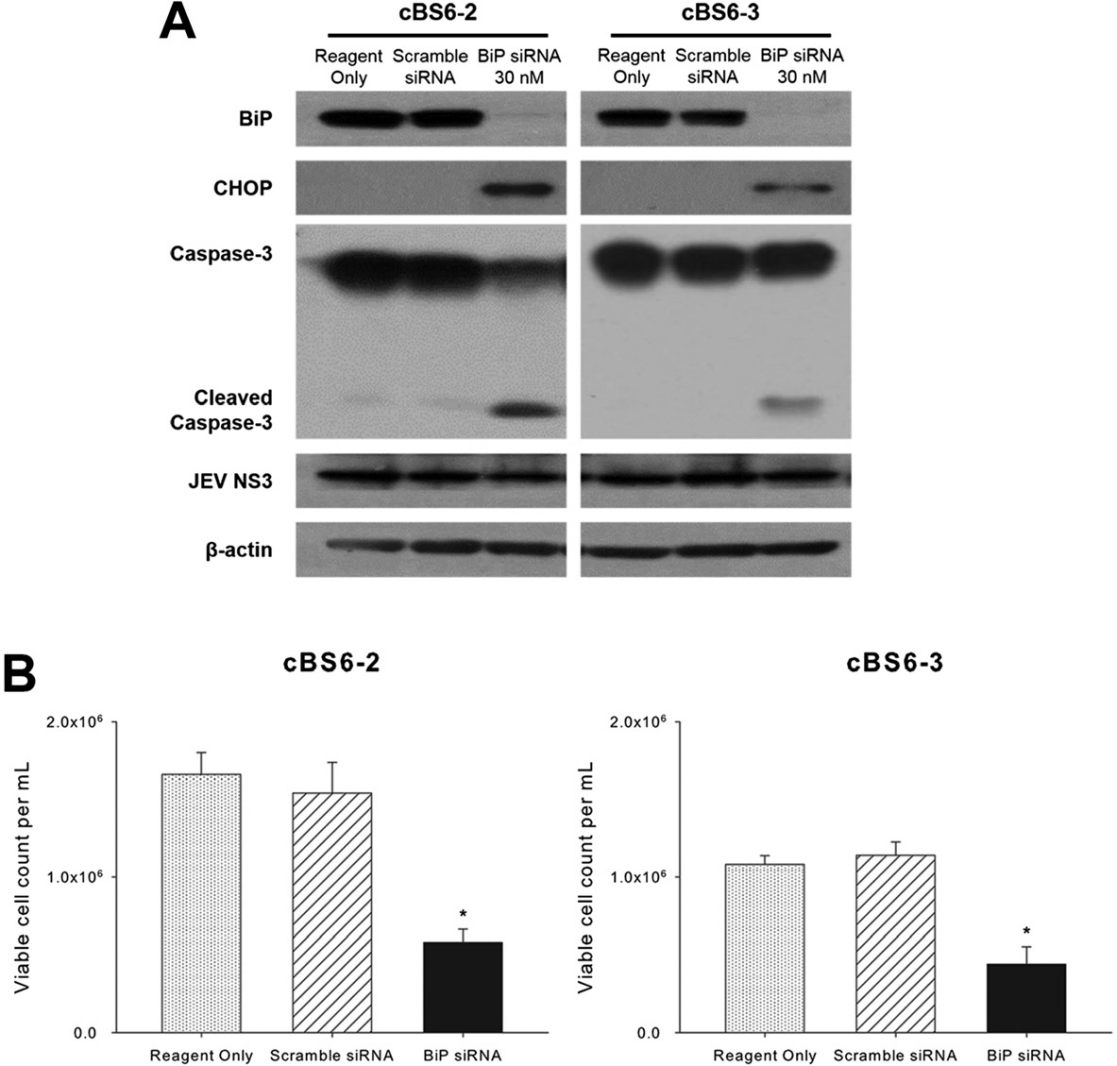
**Effects of BiP silencing on persistently JEV-infected cell clones. (A)** Persistently JEV-infected cell clones, cBS6-2 and cBS6-3, were transfected with specific siRNA for BiP or non-specific scramble siRNA at the indicated concentration for 40 hr. The cell lysates were collected to measure the expression levels of BiP, CHOP, caspase-3, JEV NS3, and β-actin as an internal control. **(B)** The number of viable cells from the sample of **(A)** was determined using trypan blue exclusion. *P < 0.05.

The cBS6-2 and cBS6-3 cells were transfected with specific siRNA for BiP and harvested 40 hr later. The effect of BiP silencing was examined by Western blotting with antibodies against BiP, CHOP, caspase-3, and JEV NS3. The expression of BiP was suppressed almost completely by siBiP in both cBS6-2 and cBS6-3 cells, while the CHOP expression was clearly induced (Figure 4A). In addition, the numbers of viable cells decreased significantly in both cell lines according to cleavage of caspase-3 (Figure 4A and B). The results suggest that the constant overexpression of BiP in the JEV persistently infected cells somehow holds back CHOP expression, resulting in the prevention of virus-induced apoptosis. This observation is consistent with reports that the inhibition of CHOP guarantees a higher survival rate both *in vivo* and *in vitro* even though CHOP is not the sole factor promoting cell death undergoing ER stress [38,39]. Furthermore, these results revealed that resistance against virus-induced apoptosis of the cBS6-2 and cBS6-3 cells did not result from the lower level of virus replication efficiency; rather it was ascribable primarily to the active participation of cellular factors in the UPR.

## Conclusions

In conclusion, BHK-21 cells with JEV persistent infection strive against virus-induced apoptosis through constant up-regulation of BiP expression, a key chaperone involved in ER stress. As demonstrated in tunicamycin treatment, these cells maintained their capacity to decide their own cell death fate by inducing CHOP, although some of the UPR factors relaying the BiP-PERK-ATF4 arm appeared to be impaired.

In a previous study, we successfully established JEV persistent infection in several mammalian cells in the presence of DI particle generation [27]. Certain modifications in the genetic makeup of helper JEV could also play a role in the development and maintenance of the viral persistent infection [27]. Therefore, the observations made in this experiment demonstrate that both the compromised virus replication capacity and certain cellular factors, such as the UPR factors, also participate in establishing viral persistent infection in mammalian cells.

Our study highlights the importance of certain host cell factors in ER stress-signaling pathways for JEV persistence by utilizing an *in vitro* model for the first time. This work provides new insight into the complex mechanism of viral persistence and potentially contributes to developing useful agents and tools for therapeutic intervention in the clinical sequelae of Japanese encephalitis.

## Materials and methods

### Cells and virus

Baby hamster kidney-21 (BHK-21; Korea Cell Line Bank) cells were maintained in a minimum essential medium (MEM; Gibco) containing 5% FBS (Gibco) and 100 units of penicillin-streptomycin (Gibco) mL^−1^. The persistently JEV-infected BHK-21 cells have been described previously [27]. Two cell clones, cBS6-2 and cBS6-3, were chosen for use. All cells were grown at 37°C in a 5% CO_2_ incubator. JEV K94P05 strain (provided by the Korean National Institute of Health) was employed throughout this study. Propagation of the virus was carried out in BHK-21 at 37°C in MEM supplemented with 5% FBS for 72 hr. After infection, the virus-containing supernatant was collected and centrifuged to remove cell debris, and then stored at −72°C.

### Plaque-forming assay

The virus was inoculated on a monolayer of BHK-21 cells in 35 mm plates; an overlay medium was applied containing 5% FBS, 1% penicillin-streptomycin (10,000 U), 15% 5× MEM, 61% 1× MEM, and 1% agarose. After 3 to 4 days incubation, the cells were fixed with 3.7% formaldehyde in phosphate buffered saline (PBS) for 2 hr and stained with 0.1% crystal violet. To titrate the intracellular virus particles, the infected cells were washed with PBS, trypsinized, and resuspended in 1 ml of MEM. After three times of freeze and thaw cycles, the cell debris was pelleted before collecting the virus-containing supernatant. The virus titer was determined by a plaque-forming assay.

### Flow cytometry

To analyze apoptosis, an annexin V-fluorescein isothiocyanate (FITC) and PI double-staining method (MEBCYTO Apoptosis kit; MBL) was used according to the manufacturer’s protocol. After the adherent cells were harvested, they were re-suspended in binding buffer, and 5 μL annexin V-FITC and 1.5 μL PI were added to the cell samples. The mixtures were incubated for 15 min in the dark at room temperature and then analyzed by flow cytometry (BD FACSCalibur).

### Antibodies and reagent

Tunicamycin (Sigma-Aldrich) was dissolved in DMSO. Rabbit anti-NS3 antibody was kindly provided by Professor Radhakrishnan Padmanabhan (Georgetown University, USA). Antibodies against p-PERK, total PERK, p-eIF2α, total eIF2α, BiP, and caspase-3 were purchased from Cell Signaling Technology. Antibodies against CHOP and Bcl-2 were purchased from Santa Cruz Biotechnology. Anti-ATF4 antibody and Anti-β-Actin antibody were sourced from Abcam and NeoMarkers, respectively. HRP-conjugated goat anti-mouse antibody and HRP-conjugated goat anti-rabbit antibody were obtained from Molecular Probes and Invitrogen, respectively.

### RNA interference

The BHK-21 cells, cBS6-2 and cBS6-3, were seeded in 6-well plates and grown to 50% confluence. siCHOP, siBiP, and scramble siRNA were purchased from Santa Cruz. Transfection of siRNA was conducted using jetPRIME™ (Polyplus-transfection) according to the manufacturer’s instructions. The BHK-21 cells were infected with JEV at 24 hr post-transfection. The cells and the supernatant were harvested at 48 hr post-infection, and the PI cell clones were harvested at 40 hr post-transfection for further analysis. The collected supernatant was used for the quantification of the viral production, and viable cells were counted using trypan blue exclusion.

### SDS-PAGE and Western blot analysis

For total protein extraction, virus- or mock-infected cells in monolayers were washed with cold PBS and then lysed in ice-cold M-PER buffer (Pierce) with a cocktail of protease inhibitors (Roche). Proteins were separated with 7.5% or 12% gradient PAGE using the Gradi-Gel™ gradient analysis kit (Elpis biotech); they were subsequently transferred to PVDF membrane (Millipore). The membrane was blocked with 5% skim milk in TBST. Primary antibodies were incubated at 4°C with membrane overnight in 3% skim milk in TBST or 3% BSA in TBST. After primary incubation, the membrane was washed in TBST once for 5 min and three times for 10 min; it then was incubated with secondary antibodies in 3% skim milk in TBST at room temperature for 2 hr. The membrane was washed again three times in TBST for 10 min, and the proteins were detected with an enhanced luminol-based chemiluminescent detection kit (AbFrontier) according to the manufacturer’s instructions.

### Densitometry

The intensities of bands from Western blot analysis were quantified using the ImageJ program (National Institutes of Health) according to the developer’s instructions.

### Statistical analysis

The data were presented as mean ± standard deviations (SD) of three independent experiments. The differences between groups were assessed by Student’s *t* test. A *P* value < 0.05 was considered statistically significant. All statistical analyses were performed using SPSS.

## Additional file

Additional file 1: Figure S1. Modulation of BiP and CHOP expression in the persistently JEV-infected cell clones. Protein samples were collected from BHK-21 cells infected with JEV at an MOI of 1 at 0 to 36 hr p.i., and from persistently JEV-infected (PI) cell clones cBS6-2 and cBS6-3. Cell lysates were analyzed by Western blotting for CHOP, BiP, JEV NS3, and the internal control β-actin. Band intensities for BiP and NS3 were determined by densitometry and normalized to those for β-actin.
