# Supplementary figures and images for: Constant up-regulation of BiP/GRP78 expression prevents virus-induced apoptosis in BHK-21 cells with Japanese encephalitis virus persistent infection
Source: Virol J. 2015 Feb 26;12:32. doi: 10.1186/s12985-015-0269-5 (PMC4352245; doi:10.1186/s12985-015-0269-5)

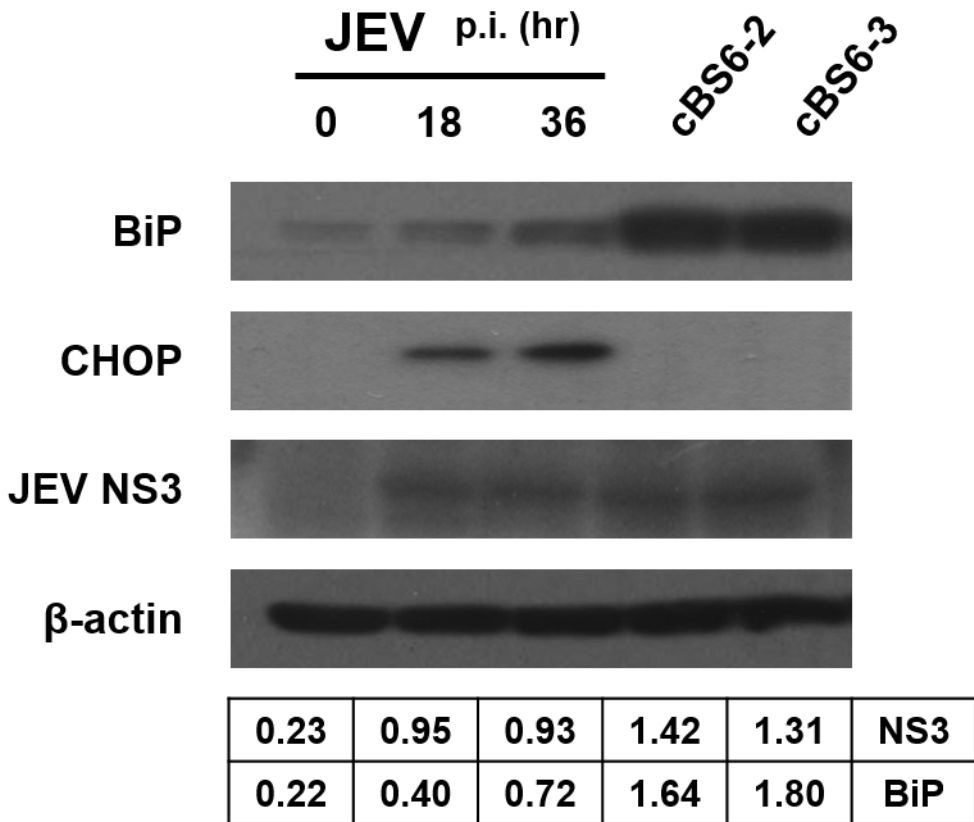

Supplement: Additional file 1: Figure S1. — Modulation of BiP and CHOP expression in the persistently JEV-infected cell clones. Protein samples were collected from BHK-21 cells infected with JEV at an MOI of 1 at 0 to 36 hr p.i., and from persistently JEV-infected (PI) cell clones cBS6-2 and cBS6-3. Cell lysates were analyzed by Western blotting for CHOP, BiP, JEV NS3, and the internal control β-actin. Band intensities for BiP and NS3 were determined by densitometry and normalized to those for β-actin. [file 12985_2015_269_MOESM1_ESM.pdf]
